# Ins and outs of AlphaFold2 transmembrane protein structure predictions

**DOI:** 10.1007/s00018-021-04112-1

**Published:** 2022-01-15

**Authors:** Tamás Hegedűs, Markus Geisler, Gergely László Lukács, Bianka Farkas

**Affiliations:** 1 Department of Biophysics and Radiation Biology, Semmelweis University, Budapest, Hungary; 2 TKI, Eötvös Loránd Research Network, Budapest, Hungary; 3 Department of Biology, University of Fribourg, Fribourg, Switzerland; 4 Department of Physiology and Biochemistry, McGill University, Montreal, QC Canada; 5 Faculty of Information Technology and Bionics, Pázmány Péter Catholic University, Budapest, Hungary

**Keywords:** Structure prediction, Transmembrane proteins, AlphaFold2, Bioinformatics

## Abstract

Transmembrane (TM) proteins are major drug targets, but their structure determination, a prerequisite for rational drug design, remains challenging. Recently, the DeepMind’s AlphaFold2 machine learning method greatly expanded the structural coverage of sequences with high accuracy. Since the employed algorithm did not take specific properties of TM proteins into account, the reliability of the generated TM structures should be assessed. Therefore, we quantitatively investigated the quality of structures at genome scales, at the level of ABC protein superfamily folds and for specific membrane proteins (e.g. dimer modeling and stability in molecular dynamics simulations). We tested template-free structure prediction with a challenging TM CASP14 target and several TM protein structures published after AlphaFold2 training. Our results suggest that AlphaFold2 performs well in the case of TM proteins and its neural network is not overfitted. We conclude that cautious applications of AlphaFold2 structural models will advance TM protein-associated studies at an unexpected level.

**Supplementary Information:**

The online version contains supplementary material available at 10.1007/s00018-021-04112-1.

## Introduction

Although enormous resources were devoted to predict protein structures for many decades, building a protein structure from its sequence remained a challenging task [1]. There was a change at the 13th Critical Assessment of Protein Structure Prediction (CASP13) competition [2] when the neural network-based approach, AlphaFold excelled. The improved version, AlphaFold2 (AF2) achieved an accuracy level much higher than other predictors at CASP14 [3, 4]. Importantly, DeepMind released their code with deep learning models and deposited AF2-predicted structures for the human [5] and 20 other proteomes in collaboration with EBI (https://alphafold.ebi.ac.uk). Moreover, to ease the running of predictions for researchers, DeepMind [6] and community Google Collaboration notebooks [7] have been generated, albeit applying some simplifications.

AlphaFold2 was trained using multiple sequence alignments (MSA) and experimental protein structures deposited before 2018-04-30. Five different models were trained (e.g. with different random seeds, with or without structural templates) to promote an increased diversity in structure predictions [6]. The input for prediction is the sequence of a single protein chain, used for MSA generation and structural template search. The quality of the resulted structural models is characterized by the mean of per residue pLDDT (predicted Local Distance Difference Test) score (which takes values between 0 and 100, the higher value is better) and the structures are ranked accordingly [3]. The pLDDT confidence measure predicts the accuracy of the Cα Local Distance Difference Test (lDDT-Cα) for the corresponding prediction. Although this means that the high accuracy and reliability of AF2 observed in CASP14 can be transferred to predicting the structure of any protein sequences (or whole proteomes) [3, 5], this has not been validated yet and scientists do not have a clear indication how well AF2-predicted structures can be trusted. Moreover, AlphaFold2 structural prediction of transmembrane proteins is treated with skepticism, as it remain challenging by both experimental and computational methods, especially because AlphaFold2 was not tuned for TM proteins. It is also not known, whether the structural model with the highest pLDDT score always corresponds to the native structure. To tackle these issues, we investigated if AF2-predicted human α-helical TM protein structures exhibit correctly located TM regions. To demonstrate at a higher resolution that the predicted TM folds are native, we compared predicted structures of the ATP Binding Cassette (ABC) superfamily from the AF2-predicted 21 proteomes to existing experimental ABC folds.

ABC proteins play a role in important cellular processes in all types of organisms and most of them transport substrates through the cell membrane in an ATP-dependent manner [8–10]. ABCC7/CFTR is a special member, which is an ATP-gated chloride channel and includes a long intrinsically disordered regulatory R domain [11, 12]. The functional form of ABC proteins is built from two highly conservative nucleotide-binding domains (NBDs) and two transmembrane domains (TMDs) which can be encoded in one or separate peptide chains. The low conservation of their TMDs are related to diverse functions and their currently known TM folds are also structurally divergent and can be classified into eight groups (Pgp-, ABCG2-, MalFG-, BtuC-, EcfT-, LptFG-, MacB-, and MlaE-like folds) [13, 14]. Our results suggest that AlphaFold2 provides protein structures for transmembrane proteins as reliable as for soluble proteins and can help to solve many issues associated with transmembrane protein structures.

## Results

### Transmembrane topology assignments in AlphaFold2 structures

**Fig. 1:**
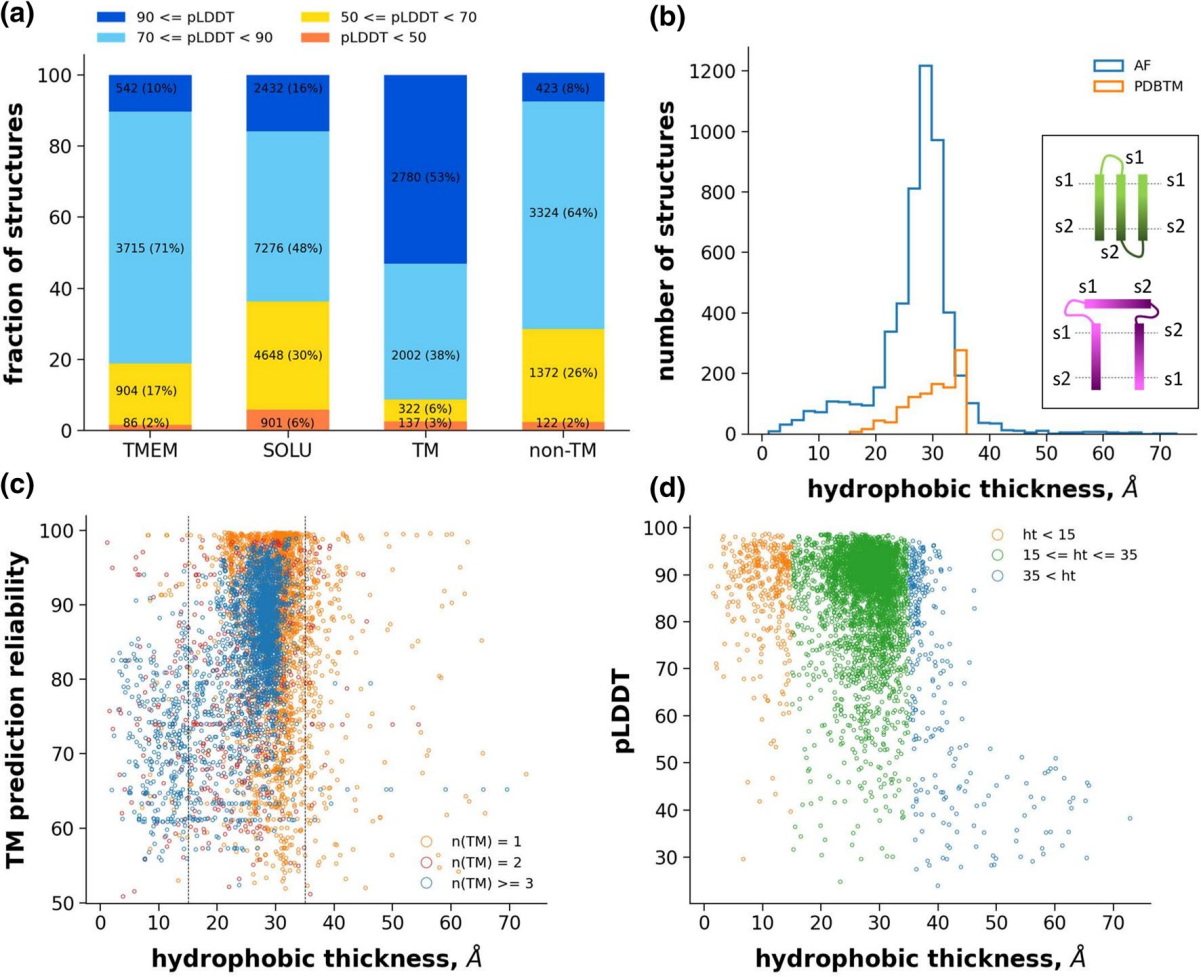
Quantitative analysis of human AF TM structures. (**a**) Mean pLDDT scores were calculated for human transmembrane (TMEM), soluble (SOLU), TM regions of TM proteins, and non-TM regions of the same proteins. The fraction of structures in reliability ranges, used in the human proteome AlphaFold2 paper [5], are shown. (**b**) The hydrophobic thickness was calculated for human TM proteins as the distance between the center of geometry of Cα atoms of side1 and side2 of transmembrane helices. TM helices of AF2-structures were selected based on CCTOP predictions. The hydrophobic thickness of experimental structures was collected from PDBTM. The inset demonstrates how the distance calculation can be effected by a topology in the case of incorrectly built AF2 structures (purple; correct structure: green; s1 and s2: side1 and side2). (**c**) The hydrophobic thickness of each protein and the corresponding CCTOP reliability scores are shown. (**d**) The hydrophobic thickness of each protein and the corresponding pLDDT scores were plotted

First, pLDDT score distribution for soluble and transmembrane proteins were compared. We split the human AF2 structures to these two groups using the HTP (Human Transmembrane Proteome) database [15], calculated the mean pLDDT score for each protein, and plotted their distribution (Fig. 1a and Fig. S1). Mean pLDDT values were also calculated separately for the TM and non-TM regions of transmembrane proteins. Intriguingly, soluble proteins exhibited a broader distribution and a significant area at lower pLDDT values compared to TM proteins. This was unexpected, since the majority of the AlphaFold2 learning set inherently included more soluble protein templates and the algorithm was not tuned for transmembrane proteins. However, correlation between low pLDDT values and disordered segments was observed [5], thus our observation suggested that more soluble proteins possess disordered regions than TM proteins. Interestingly, a very large portion of TM regions (53%) were predicted with high pLDDT scores (> 90) (Fig. 1a) indicating that AF2 captured the rules governing protein structures within the hydrophobic region.

Next, we compared the spatial localization of TM helices in AF2 structures if helix orientation corresponds with rational and physiological orientation in a lipid bilayer slab using the Constrained Consensus Topology prediction (CCTOP) software [16], which includes information from both experimental and computational sources. We separated the start and end positions of predicted TM helices to two residue sets according to their localization relative to the opposite sides of the bilayer. The distance between the center of geometry of the two sets were calculated and its distribution is plotted (Fig. 1b). The majority of the membrane thickness values were in the range between 20 and 35 Å, which is in the range of the hydrophobic region thickness. To support this finding with experimental data, the hydrophobic thickness of experimentally determined human transmembrane protein structures was retrieved from the PDBTM database [17]. The AF2 and experimental distribution largely overlapped (Fig. 1b). These observations suggested that hydrophobic thickness values below 15 Å or above 35 Å may indicate an erroneous AF2 structure (725 out of 5,952, 12%, Table S1). An inaccurate TM topology prediction of CCTOP may provide an outlier hydrophobic thickness in the case of a correct AF2-predicted structure. The CCTOP reliability versus thickness plot (Fig. 1c) indicated that the topology of most proteins, whose AF2-predicted structure exhibited hydrophobic thickness within the 15–35 Å regime, was predicted with high reliability. Structures with lower hydrophobic thickness values and high CCTOP reliability were likely inaccurately predicted by AlphaFold2, while structure predictions with lower thickness and lower CCTOP scores were located in the twilight zone. Intriguingly, we observed that some of these entries may have low topology reliability because of their existence in protein–protein complexes, but AF2 predicted the monomeric form correctly (Fig. S2). This suggests that AF2 may also be used to identify and aid the correction of improper membrane topology predictions.

We also investigated the distribution of pLDDT scores versus hydrophobic thickness (Fig. 1d). This plot indicated that AF2 structures with non-physiological thickness values can process very high pLDDT scores, consequently, these scores alone may be insufficient to select correct TM structures in blind predictions.

### Helix packing in AF2-predicted ABC models overlaps with experimental folds

**Fig. 2:**
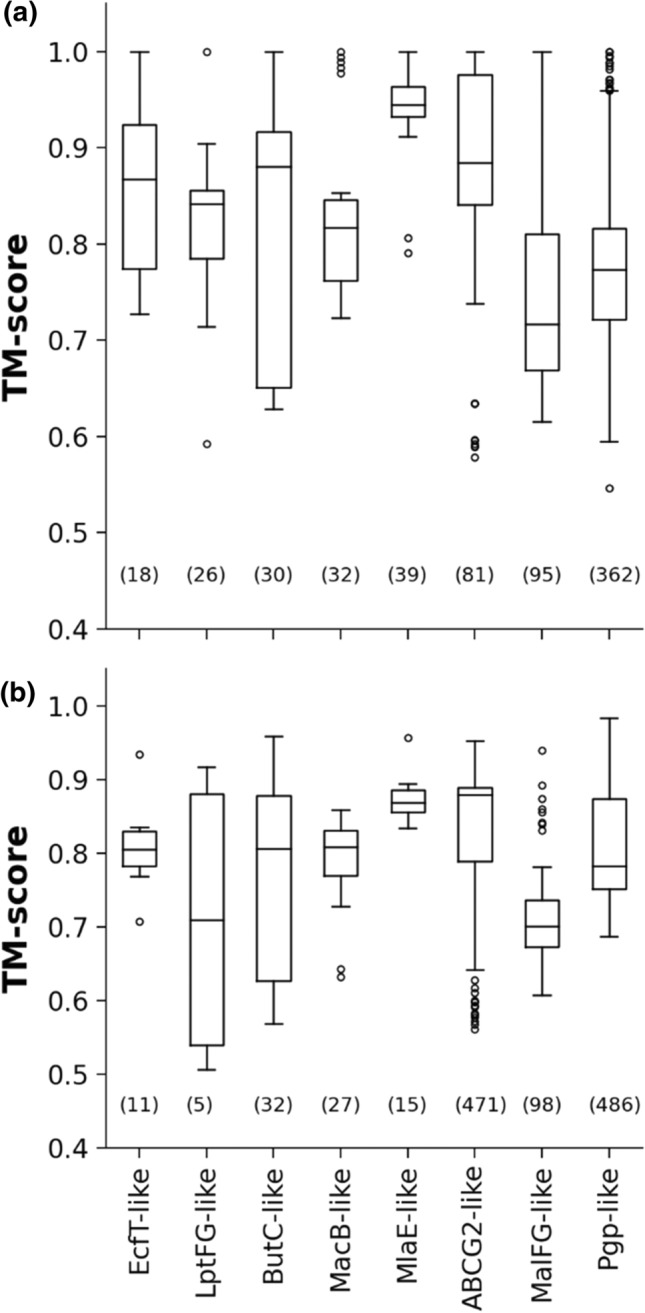
All AF2-predicted ABC structures exhibit valid ABC TM folds. (**a**) The best TM-score for every experimental ABC TM structure and ABC reference fold comparisons are shown in the boxplot, grouped into ABC fold families, sorted by the total number of included transmembrane chains. Numbers indicate the sum of chains in experimental structures matching an ABC reference fold. Only MlaE fold family does not contain experimental structures released before 2018-04-30. (**b**) The same plot was generated for ABC protein structures from the 21 proteomes predicted by AF2. The number of matched structures within a fold family is indicated in parenthesis. A TM-score above 0.5 indicates that the compared structure and the reference fold exhibits the same architecture

To assess AF2 TM protein predictions at a higher resolution, we aimed to compare AF2-built ABC TM folds with experimentally determined folds. Structures of ABC superfamily members are a reasonable choice to investigate AlphaFold2 performance on TM proteins, since the currently available PDB entries, which include 675 chains with ABC transmembrane domains, are diverse and can be classified into 8 different structural folds (Fig. S3) [13, 14]. We characterized the similarity of each ABC transmembrane domain to every ABC reference fold using the Template Modeling score (TM-score) [18, 19] (Fig. 2a). If comparison of two structures results in a TM-score below 0.3 then they are structurally unrelated, while a TM-score above 0.5 indicates identical folds [19]. The range between 0.3 and 0.5 is the twilight zone. Each target transmembrane domain was classified according to the best match to an ABC reference fold and the TM-scores were above 0.5 in all cases. The observed variation of scores among these experimental ABC structures originated from differences in conformations (e.g. apo and ATP-bound structures).

In the next step, we selected ABC structures from the 21 proteomes with AF2 predictions by a stringent PFAM search, which was performed with 28 PFAM Hidden Markov Models (Table S2) that resulted in 1137 hits. For assessing the similarity of structures to the eight selected reference folds, we calculated TM-scores between the AF2-predicted transmembrane ABC structures and the reference structures. The best out of eight scores were saved for each structure. We found that all TM-score values were above 0.5 (Fig. 2b). One outlier protein (Q2G2E2), which matched the YitT_transmembrane PFAM entry, was somewhat similar to the aquaporin/GlpF fold (e.g. PDBID: 1fx8) suggesting that the YitT_transporter PFAM entry is wrongly classified. Indeed, this protein belongs to the non-ABC, Novobiocin Exporter (NbcE) Family based on the Transport Classification Database [20].

Some of the predicted ABC structures included additional N-terminal TM-like helices, which were somewhat distant from the core TM domain and likely are membrane-associated regions, such as the L0/Lasso motif of ABCC proteins [21–23]. In many cases, membrane-associated regions, loops, and mobile segments not resolved in experimental structures have been rationally modeled by AF2, based on visual inspection (see below and Fig. S2), thus the AF2 machine learning method may have grasped some knowledge on a lipid bilayer around TM proteins. However, in other cases, long loops with low pLDDT scores, which are likely disordered regions, were unrealistically crossing the bilayer region. Those in our eyes are not negatively affecting AF2 predictions and were thus not considered as an issue, since the localization of disordered regions also cannot be trusted in the case of AF2-predicted soluble protein structures.

### Prediction of challenging and novel transmembrane folds

Importantly, the above and any retrospective analysis of AF2 predictions are limited by the fact that a significant portion of the AF2-predicted (transmembrane) protein structures deposited at EBI have corresponding experimental structures with either the same sequence or a homologous sequence, either included in the AF2 training set (up to 2018–04-30) or used as templates during prediction runs (up to mid of 2020). Therefore, we selected the challenging TM target of CASP14 (T1024, LmrP, PDBID: 6t1z released on 2019–10-07), which possessed homologous structures, and novel TM folds that were also released after 2018–04-30 for characterizing AF2 performance.

**Fig. 3:**
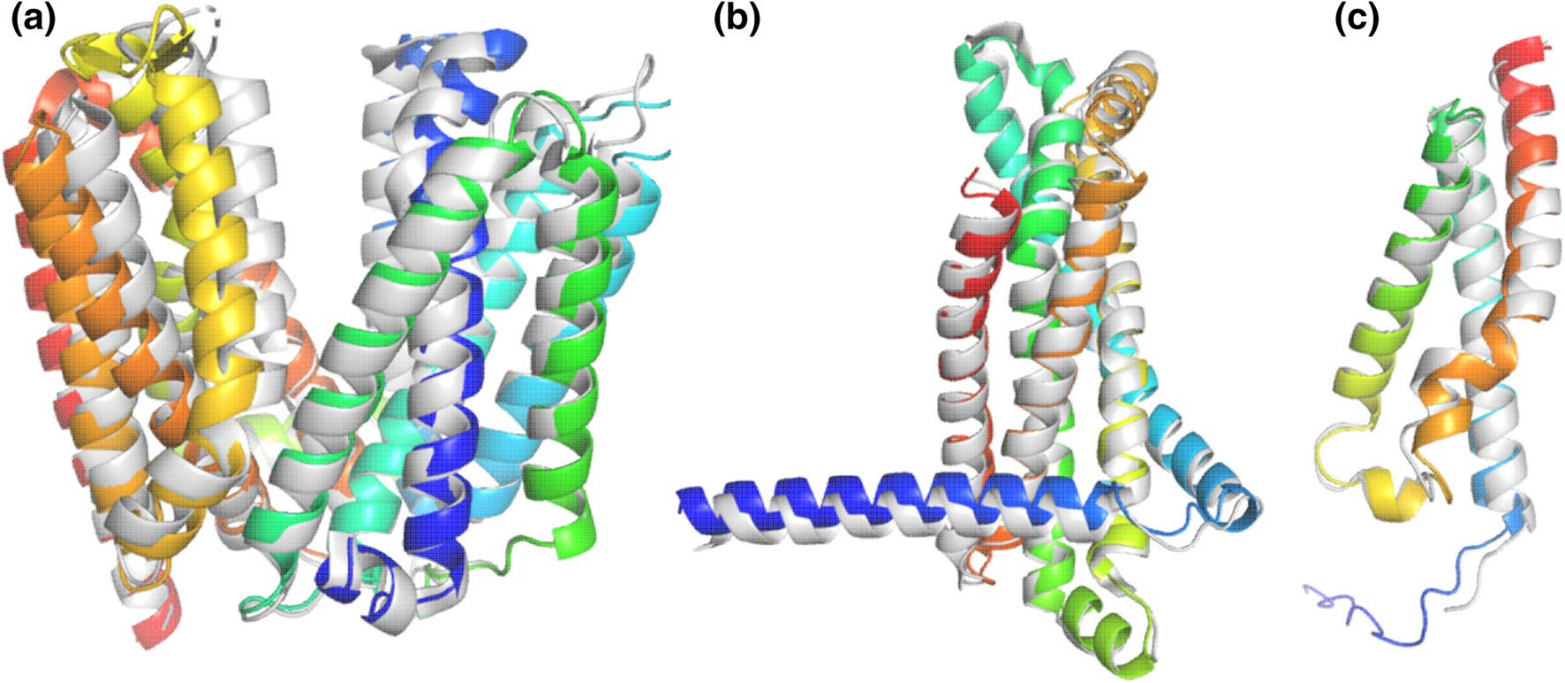
Blind transmembrane fold predictions. The AF2-predicted structures (blue to red: N- to C-termini) of the challenging CASP14 TM target, LmrP (**a**), the MlaE (**b**), and the EMC6 (c) exhibit perfect alignments with their experimental structures (gray) 6t1z, 7ch0, and 6ww7, respectively. None of these experimental structures were published before 2018-04-30. Only structures with a sequence homologous to LmrP were in the AF2 training set

The prediction of the T1024 target, ranked #43 with GDT_TS score and RMSD of 60.29 and 5.61 Å, respectively (#1 by Arne Elofsson: 63.3 and 3.74 Å). However, LmrP has a hinge region that effects predictions and AF2 likely produced a functional conformation different from that observed in the 6t1z structure, supported by distance restraints from double electron–electron resonance spectroscopy [24]. Since the AF2 LmrP model submitted to CASP14 was created with an earlier version of AlphaFold [25], we rerun the LmrP prediction with disabled template usage. The top model exhibited 82.82, 1.74 Å, and 0.92 GDT_TS, RMSD and TM-score, respectively, when compared to 6t1z (Fig. 3a). These observations suggest that AF2 prediction of flexible targets should be interpreted carefully and AF2 may be utilized to discover novel conformations related to different functional states.

**Fig. 4:**
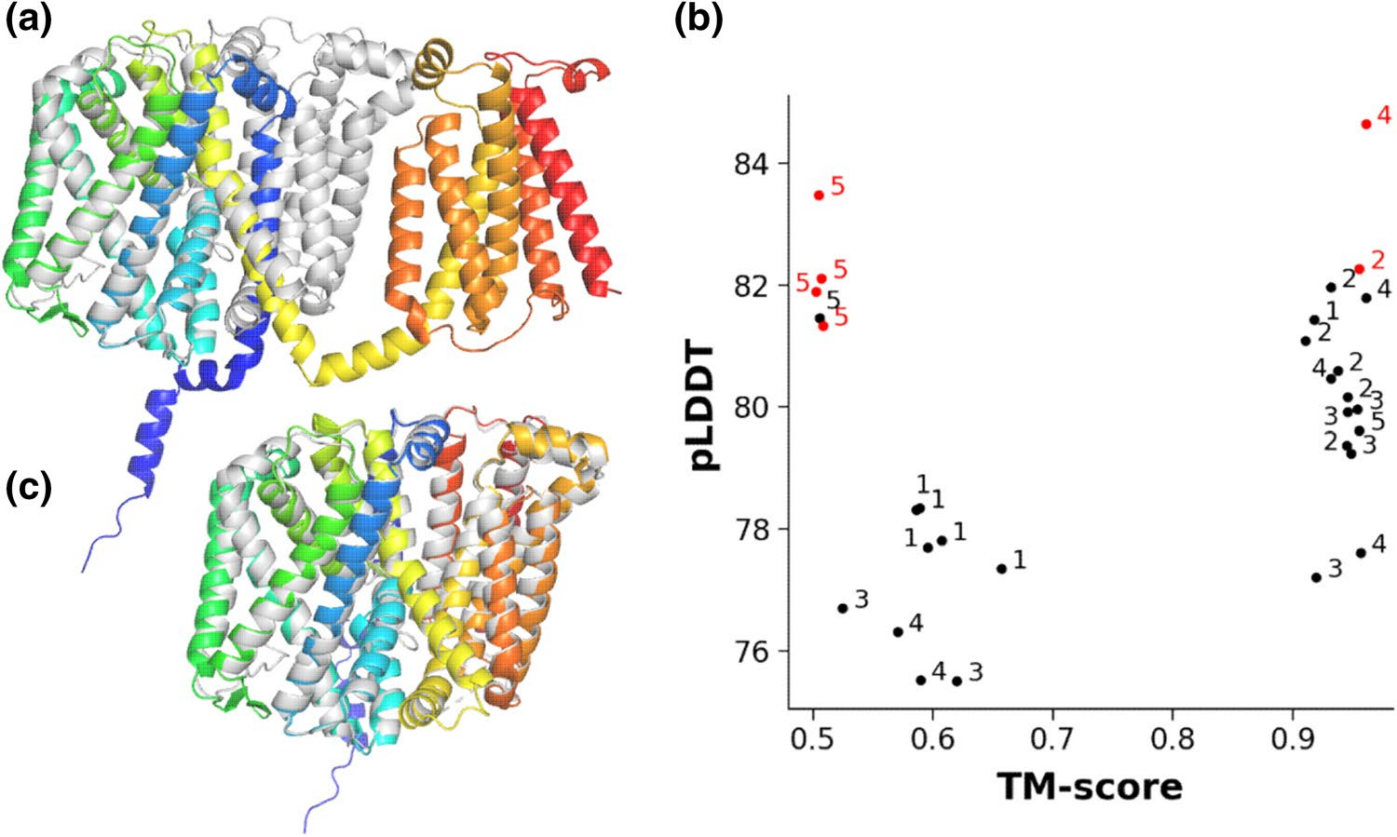
AF2 predicts two conformations of a new transmembrane fold. (**a**) The top AF2-prediction of the novel MprF TM fold (blue to red: N- to C-termini) aligned to the experimental structure 7duw (gray). (**b**) pLDDT and TM-score values, calculated for every structural model from six runs, were plotted. Numbers (1–5) indicate the corresponding AF2 models. Red points were the top ranked hits from a given run. (**c**) Structural alignment of the prediction with the best pLDDT score (blue to red) and experimental structure (gray). 7duw and any other structure homologous to MprF were not included in the AF2 training set

In the next step, we performed extensive literature, SCOP, and PFAM searches to identify transmembrane protein structures or their homologous structures, which were not inserted into the AF2 training set. We found the ABC transmembrane MlaE-like fold (7cge, 7ch0, and 7cgn were released on 2020-09-09; 7ch7 was released on 2021–05-19) [26], the ER membrane protein complex subunit sixfold (EMC6, PDBIDs: 6wb9, 6ww7, 6z3w, 7ado, 7adp, 7kra, and 7ktx, with the earliest release date of 2020-05-27) [27], and the MprF structure (PDBIDs: 6lvf and 7duw, released on 2021-02-03 and 2021-04-21, respectively) [28] as valid targets for blind AF2 TM protein predictions. AF2 runs without templates resulted in top models highly similar to the experimental structures of MlaE (PDBID: 7ch0, RMSD: 1.28 Å, TM-score: 0.95, Fig. 3b) or EMC6 (PDBID: 6ww7, RMSD: 0.96 Å, TM-score: 0.93, Fig. 3c). In contrast, the top prediction of the multiple peptide resistance factor (MprF) transmembrane domain sequence did not match the experimental structure (Fig. 4a). Therefore, we performed this prediction several times (*n* = 6) with different random seeds and compared the output to the transmembrane domain of 7duw using TM-score. Plotting the pLDDT scores versus TM-scores (Fig. 4b) indicated that among the 30 predicted structures the one with the best pLDDT score exhibited the highest TM-score, thus was the most similar to the target structure (Fig. 4c). Importantly, the difference in MprF conformations involves the separation of two subdomains (flippase and synthase) [29] and AF2 may have captured a functionally relevant state as in the case of LmrP.

### AF2 can provide hints for investigating ABC structure-associated questions

**Fig. 5:**
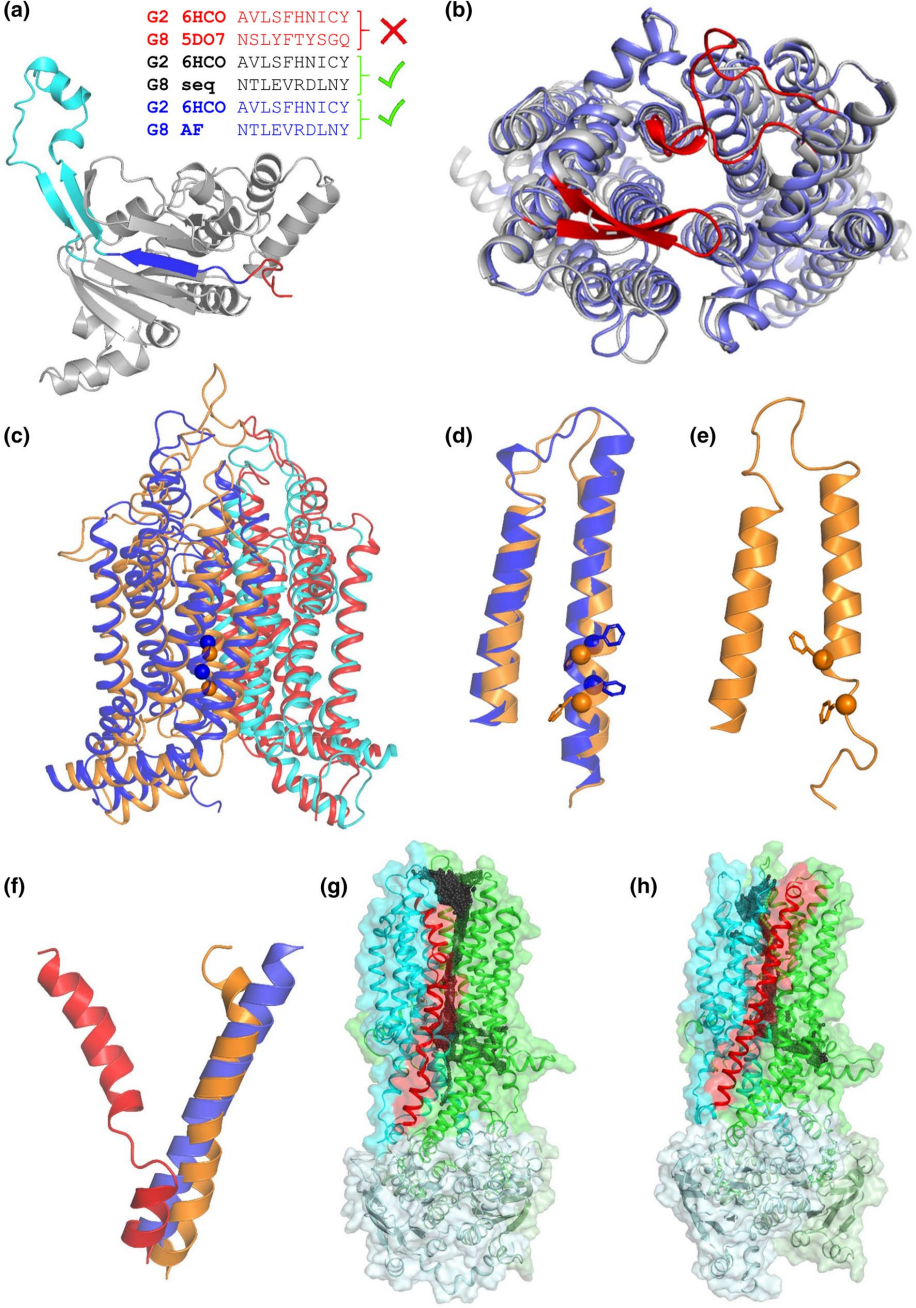
AF2 predictions and ABC structure-associated issues. (**a**) ABCG2 and ABCG8 NBD β1 strand sequence alignments generated by structural alignment of 6hco (ABCG2) and 5do7 (ABCG5/ABCG8), by ClustalW with manual adjustment of ABCG2 and ABCG8 sequences based on ABCG2 structures, and by structural alignment of ABCG2 and AF2-predicted ABCG8 NBDs. Structure: AF2 ABCG8 NBD, blue: β1 strand, red: the segment corresponding to the β1 strand in the registry shifted 5do7 NBD, cyan: gating loop or regulatory insertion. (**b**) Structural alignment of 7jr7 (gray) and AF2-predicted (blue) ABCG5/ABCG8 TM domains (top view). Non-conserved loops with low-quality predictions are red. (**c**) Aligned homology (orange: TMD1, red: TMD2) and AF2 (blue: TMD1, cyan: TMD2) models of AtABCG36. Blue and orange spheres label F589 and F592 in TM2 facing the substrate binding pocket. (**d**) The magnified view of AtABCG36 TM1 and TM2 indicates that the alignments are not shifted but that spatial localization and side chain packing differ. (**e**) TM2 in the homology model unwinds in MD simulations. (**f**) zfCFTR TM8 is kinked in PDBID:5w81 (red) along with other structures and it is straight in both MRP1-based model (orange) and AF2-predicted structure (blue). The helices are extracted from a full TM domain alignment for visualization. (**g**) Surface representation of zfCFTR (PDBID:5w81). Red: TM8, green: TMD1, cyan: TMD2, pale green: NBD1, pale cyan: NBD2, black spheres: CAVER spheres indicating channel opening towards the extracellular space and the extracellular boundary of the lipid bilayer. (**h**) Surface representation of zfCFTR with MRP1-modelled, straight TM8. No lateral opening to the extracellular membrane leaflet can be observed

To demonstrate possible contributions of AF2-predicted structural models to studies targeting membrane proteins, we assessed AF2 ABC models in various test cases. At first place, we tested half transporter ABCG proteins, which consist of an NBD and a TMD in a polypeptide chain and function in homodimeric or heterodimeric complexes [14]. The first experimentally determined ABCG2-like fold was the X-ray structure of the ABCG5/ABCG8 heterodimer (PDBID: 5do7) published in 2016 [30]. Our first observation with the AF2-generated ABCG8 structure was regarding its soluble NBD. After the publication of the first ABCG2 structure [31], structural alignment and sequence analysis indicated a registry shift in the first β-strand of ABCG8 NBD (Fig. 5a) that happened because of the low resolution of this region. Although the 5do7 structure was in the AF2 training set and was present in the pdb70 template database, the AF2-predicted ABCG8 structure deposited at EBI did not have this error (Fig. 5a). An ABCG5/ABCG8 structure with a correct registry was also released on 2021-04-07 (PDBID: 7jr7 [32]), but AF2 template search for building models deposited at EBI used pdb70 downloaded on 2021-02-10 [5].

To assess ABCG5/ABCG8 transmembrane domain (TMD) predictions, we ran AF2 without application of templates. First, the ABCG5 TMD predictions were of exceptionally good quality regarding the RMSD (root mean square deviation) and TM-score values of 0.61 Å and 0.94, respectively, when compared to the ABCG5 chain in the 7jr7 structure. Second, we investigated ABCG5/ABCG8 heterodimer predictions. Since only single chains can be submitted to AlphaFold2, we concatenated the two sequences with a part of the CFTR R domain sequence (a.a. 675-800). This disordered sequence was sufficiently long not to constrain the conformational space of the dimer and did not exhibit strong intramolecular interactions even in its native, AF2-predicted structural environment (Fig. S4). The predicted TMD dimer exhibited 2.18 Å RMSD and its individual chains showed 0.98 and 0.96 TM-score values when compared with the 7jr7 structure (Fig. 5b).

To investigate if AlphaFold2 can distinguish between intra- and intermolecular interactions in the case of homomeric complexes, we performed a prediction with ABCG2, which forms homodimers [33]. The complex of the two identical TMDs was also predicted exceptionally well (2.42 Å RMSD and 0.9 TM-score when compared to PDBID: 6vxf). Interestingly, cysteine residues forming intra- and intermolecular disulfide bonds were close to each other (Fig. S5).

We also examined how AF2 structural models can supplement or replace homology models in molecular dynamics (MD) simulations. The TM regions of distant ABC proteins exhibit low sequence conservation with good accordance of their dissimilar functions and substrates. However, their folds in a family are highly conserved, thus homology modeling can provide high-quality models [34–37]. We chose AtABCG36/PEN3/PDR8 [38] from the model plant *Arabidopsis thaliana*, which is a well-investigated full transporter of the ABCG subclass for that no structures yet exist. When the homology model exhibiting two ABCG2-like TMDs (Fig. 5c) was inserted into a membrane bilayer and subjected to a 50 ns long MD simulation, one portion of an α-helix, which is part of the central drug binding pocket, exhibited fast unfolding (~ 10 ns) in an equilibrium MD simulation. Then, the AF2-predicted AtABCG36 structure under the same conditions remained stable in a 500 ns long MD simulation (Fig. S6). However, one should be careful with simulations using AI-based structural models, since their conformation may be kinetically trapped into a specific state, inhibiting the study of conformational changes [39].

The CFTR/ABCC7 chloride channel is also a member of the ABC superfamily with a Pgp-like fold. The functional mechanism of this protein is of interest, since some mutations effect channel gating and cause cystic fibrosis [40]. One of its structures was determined using cryo-EM under activating condition, in the presence of ATP and phosphorylation, but the extracellular pore of the channel remained in a closed state, most likely due to a kink in TM8, corresponding to an unwound segment in the transmembrane region [41] (Fig. 5f). This kink is present in most CFTR structures (PDBIDs: 5uak, 5uar, 5o2p, 5w81, 6msm, and 6o1v) [41–44]. However, the kink is absent from the chicken CFTR structure (PDBIDs: 6d3s and 6d3r) [45] and such a conformation has not been detected in other ABC structures. We performed equilibrium simulations with the 5w81 structure [12] to detect channel opening, but appearance of tunnels with sufficient diameter to pass chloride ions were rare events and was observed only once out of 22 simulations (6 × 100 ns + 16 × 35 ns, 427/116,000 frames, 0.36%). Intriguingly, many of the conformations provided a tunnel opened towards lipid molecules of the extracellular membrane leaflet (Fig. 5g). After correcting the kink by homology modelling based on the MRP1 structure (PDBID: 5uj9) (Fig. 5f), opening of the extracellular pore could be observed in five out of six simulations at a higher probability (6 × 100 ns, 2245/60,000 frames, 3.74%). Remarkably, modeling CFTR TMDs using AlphaFold2 without CFTR or any templates resulted in a conformation similar to that of MRP1 with a straight TM8 helix (Fig. 5f, h). Since TM8 has been suggested to be flexible regarding to its membrane embedment [46], it is likely sensitive to its environment and based on the functional assays and the structure determination protocol [42], the detergent added in the last step (3 mM fluorinated Fos-Choline-8) likely biased the experimental structure.

## Discussion and conclusions

We demonstrated that at least ~ 90% of the AF2-predicted TM structures of the human proteome represented membrane-protein like structures, using the most available and reliable measure, the location of TM helices from consensus predictions and experimental structures, for assessing TM protein structure quality at a large scale. Since the pLDDT score distribution did not shift much to lower values compared to soluble proteins (Fig. S1), it is likely valid to state that AF2 predicts TM proteins as well as soluble proteins. However, predicted TM structures with low hydrophobic thickness and high pLDDT score (Fig. 1d) suggest that evaluation depending solely on pLDDT score may not be sufficient to select the best AF2-predicted model, at least in the case of TM proteins. A similar conclusion was drawn comparing the AF2-predicted and cryo-EM structures of the pump-like channelrhodopsin with structural features never seen before [47]. In specific cases, resource intensive molecular dynamics simulations may be used to asses AF2 models, since MD simulations were demonstrated to reveal erroneous structural models built using either homology modelling (Fig. S6) or experimental methods [48].

A very important issue is associated with retrospective studies, including ours, which assess AlphaFold2 performance based on AF2 structures deposited at EBI. Most likely a significant portion of the predicted models can be related to experimental structures with homologous sequences, included in the AF2 training set or used as templates during model building or both. In these cases, AF2 may be considered as a highly advanced homology modelling tool, which performs an automatic but high-quality sequence alignment and provides high-quality results even in the case of target sequences with low sequence similarity to any known structures. This is a very important property of AF2 and will advance structural biology studies of TM proteins, since the hydrophobic regions are usually not highly conserved (e.g. sequence identity between ABC transmembrane domains is usually below 20–30%; ABCG2 exhibits 27% and 26% identities when compared to the closely related ABCG5 and ABCG8, respectively). For the correct interpretation of retrospective studies and evaluation of AF2 performance, it is important to implement a versioning system for AF2 models. This objective seems to be more complicated than for experimental structures, since the structure prediction depends on the version of the deep learning models, various sequence databases, and the pdb70 structure database.

Taken together, investigating AF2 performance in blind predictions requires an experimental structure, which or structures with homologous sequences were not included in the training set. In addition, the AF2 prediction of such targets should be performed without using templates. In this way, predictions for a high number of homologous sequences and their systematic comparison to corresponding structures generated with templates could be informative regarding to blind predictions and to the effect of template usage. However, this type of large-scale studies using AlphaFold2 requires high resources, likely unavailable for most academic institutes. Here, we identified three transmembrane structures qualified for fully blind AF2 predictions (Fig. 3 and Fig. 4). The outputs suggested that AlphaFold2 can be reliably used for building TM structures in a blind setup. Intriguingly, both LmrP and MrpF predictions indicated that running AF2 with different random seeds may be a valid approach to predict structures corresponding to different conformational states.

Furthermore, our results demonstrate that AlphaFold2 is a highly valuable tool in many areas of TM protein research. The correction of the register shift by AF2 in ABCG8 NBD (Fig. 5a), supports the application of AlphaFold2 in molecular replacement protocols aiding experimental structure determination [49]. In addition, screening experimental structures with their corresponding AF2 structures may detect structural errors and contribute to improving PDB database quality. Similarly, the absence of the kink in CFTR TM8 in an AF2 model predicted with disabled template usage (Fig. 5f) raises novel questions that will lead us to a deeper understanding of CFTR channel function. Importantly, our runs resulting in the corrected registry shift in ABCG8 are indications against an overfitting in the neural network behind AlphaFold2 and for overcoming memory footprints originating from training. We also demonstrated that AF2 was capable of predicting transmembrane dimer structures independently of their homo- or heteromeric nature (Fig. 5b and Fig. S5), while AF2 was not trained for multimer predictions. Though, this success may be at least partially caused by the footprint of these proteins themselves in the AF2 neural network, successful protein-peptide docking [50], when peptides were not involved in alignments, is an argument against this reasoning. Interestingly, the novel deep learning model, AlphaFold2-Multimer [51], trained for predicting protein complexes is reported to excel AlphaFold2 in heteromeric but not in homomeric predictions.

In summary, our study underscores that AlphaFold2 can provide reliable protein structures also for transmembrane proteins and perform well in many areas associated with structural analysis of TM proteins. While the artificial intelligence inside AlphaFold2 can predict valuable structural information and correct structure-related flaws (e.g. registry shift, alignments, TM topology prediction, etc.), the limited predictive power of structural models from blind predictions involving flexible regions retain experimental validation desirable.

## Methods

### Databases and associated software

AlphaFold2 structures predicted for 21 proteomes were downloaded from https://alphafold.ebi.ac.uk in July, 2021. Proteins and their structures are identified in the manuscript with their UniProt accession number. Human Transmembrane Protein database [15] (2021-06-02) was received as an XML file from http://htp.enzim.hu. The data also contained CCTOP [16] (http://cctop.enzim.ttk.mta.hu) predictions and their reliability values. The hydrophobic thickness of experimentally determined human TM protein structures was retrieved from the PDBTM database (http://pdbtm.enzim.hu, 2021-07-23) [17]. Python was used to parse their XML files.

ABC PFAM entries were identified at https://pfam.xfam.org (*n* = 28) and extracted from the Pfam-A.hmm file. The selected entries and their accession numbers are listed in Table S2. The sequence of every AF2 structure was searched using HMMER hmmsearch (http://hmmer.org) [52]. The E parameter was set to 0.001 and the match length was restricted to a minimum of 90% of the HMM profile length. The hmmsearch output was parsed using BioPython [53].

Novel structural folds for multi-pass α-helical transmembrane proteins were collected by extensive literature search (match: MprF) and by manual screening of the membrane protein selection of the SCOP database [54] (80 fold families and their subfamilies; http://scop.mrc-lmb.cam.ac.uk/term/2) and corresponding entries in the PFAM database [55] (matches: MlaE and EMC6).

### Data analysis and visualization

MDAnalysis [56] and NumPy [57] Python packages were used for calculation of mean pLDDT values and hydrophobic membrane thickness. The pLDDT value of each residue were extracted from the B-factor column of AF2 structure files. For TM thickness calculation end positions of TM helices were retrieved from HTP/CCTOP and divided into two groups representing the two sides of the membrane. Plotting was done with Matplotlib (https://matplotlib.org) [58].

TM-score was calculated with TMalign [59]. Reference ABC structures are listed and shown in Fig. S3. Their TM domains were selected manually.

Molecular visualization and RMSD calculation were performed using PyMOL (The PyMOL Molecular Graphics System, Version 2.4.0 Schrödinger, LLC). RMSD of MD trajectories was calculated with the GROMACS rms tool.

### Running AlphaFold2

AlphaFold2 was downloaded from github and installed as described (https://github.com/deepmind/alphafold) on a Debian 10 box with an AMD Ryzen Threadripper 2950X 16-Core Processor. 96 GB RAM was installed and ~ 75 GB peak usage was observed during jackhmmer run. The calculation was accelerated by an NVidia Quadro P6000 GPU with 24 GB RAM, which was almost fully utilized when the predicted sequence length was 1571. The required databases were located on two 2 TB HDD in a RAID0 setup. Typical run timings were: “features”: 25–60 min, “predict_and_compile_model_*”: 3–50 min, “relax_model_*”: 1 min—6 h based on input sequences between 290 and 1571 a.a. length.

To exclude CFTR structures as templates from predictions, we modified run_alphafold.py, docker/run_docker.py, and alphafold/data/templates.py scripts to implement a -skip function. The modified scripts can be downloaded from http://alphafold.hegelab.org. Template usage was disabled by setting –max_template_date option to 1900-01-01. Dimer predictions were run by concatenating sequences with a part of the intrinsically disordered CFTR R domain, a.a. 675–800. pLDDT scores and ranking of predicted structures were extracted from the ranking_debug.json file.

### Homology modelling

AtABCG36 (UniProt ACC: Q9XIE2) was homology modeled based on an ABCG2 homodimer structure (PDBID: 6hzm) using Modeller [60]. Sequence alignment was generated using ClustalW [61] and adjusted manually. One hundred structures were generated and the one with the best DOPE score was selected for MD simulations.

zfCFTR TM7 and TM8 was homology modeled similarly. The two helices were set for modelling based on the corresponding regions of MRP1 (PDBID: 5uj9 [23]) and the rest was kept static and based on the 5w81 zfCFTR structure.

### Molecular dynamics simulations

MD simulations with AtABCG36 were performed using GROMACS 2019 with the CHARMM36m force field [62, 63]. Simulation systems were prepared using CHARMM-GUI [64, 65]. Structural models were oriented according to the OPM (Orientations of Proteins in Membranes) database [66] and all N- and C-termini were patched with ACE (acetyl) and CT3 (N-Methylamide) groups, respectively. The proteins were inserted in a bilayer with 1:1 POPC:PLPC (1-palmitoyl-2-oleoyl-sn-glycero-3-phosphocholine: 1-palmitoyl-2-linoleoyl-sn-glycero-3-phosphocholine) in the extracellular leaflet and 45:40:10:5 POPC:PLPC:POPS:PIP2 (POPS: 1-palmitoyl-2-oleoyl-sn-glycero-3-phospho-L-serine, PIP2: phosphatidylinositol 4,5-bisphosphate) in the intracellular leaflet. Both systems with the homology model or the AF2 structure were energy minimized using the steepest descent integrator (values for max. steps 50,000 and max. force 500 kJ/mol/nm were set). Six equilibration steps, according to the standard CHARMM-GUI protocol, were applied with decreasing position restraints. In the 50 ns (homology model) and 500 ns (AF2 model) long production runs, Nosé-Hoover thermostat and Parrinello-Rahman barostat with semiisotropic coupling were employed. Time constants for the thermostat and the barostat were set to 1 picosecond and 5 picosecond, respectively. The fast smooth PME algorithm [67] and LINCS algorithm [68] were used to calculate electrostatic interactions and to constrain bonds, respectively. GROMACS rmsf tools were used to calculate RMSF (root mean square fluctuation).

Simulations with the zfCFTR structure containing the kinked TM8 have been published and the protocol and parameters were described there [12]. The structure with the straightened, MRP1-based TM8 was subjected to MD simulations using the same protocol, including the same version of GROMACS, force field, and lipid composition. Channel pathways were determined using CAVER [69] as described in [12].

### Supplementary Information

Below is the link to the electronic supplementary material.

Supplementary file1 (PDF 1620 KB)

## Data Availability

All input data are available from public resources and their accession numbers are listed.

## Code availability

Modified AlphaFold2 scripts can be downloaded from http://alphafold.hegelab.org.
